# Cloud Detection: An Assessment Study from the ESA Round Robin Exercise for PROBA-V

**DOI:** 10.3390/s20072090

**Published:** 2020-04-08

**Authors:** Umberto Amato, Anestis Antoniadis, Maria Francesca Carfora

**Affiliations:** 1 Istituto di Scienze Applicate e Sistemi Intelligenti ‘E. Caianiello’ CNR, 80131 Napoli, Italy; umberto.amato@cnr.it; 2 Laboratoire Jean Kuntzmann, Department of Statistics, Université Joseph Fourier, 38000 Grenoble, France; Anestis.Antoniadis@univ-grenoble-alpes.fr; 3 Istituto per le Applicazioni del Calcolo ‘Mauro Picone’ CNR, 80100 Napoli, Italy

**Keywords:** cloud detection, PROBA-V, statistical learning, machine learning, cumulative discriminant analysis, K-Nearest Neighbor, neural networks

## Abstract

A Round Robin exercise was implemented by ESA to compare different classification methods in detecting clouds from images taken by the PROBA-V sensor. A high-quality dataset of 1350 reflectances and Clear/Cloudy corresponding labels had been prepared by ESA in the framework of the exercise. Motivated by both the experience acquired by one of the authors in this exercise and the availability of such a reliable annotated dataset, we present a full assessment of the methodology proposed therein. Our objective is also to investigate specific issues related to cloud detection when remotely sensed images comprise only a few spectral bands in the visible and near-infrared. For this purpose, we consider a bunch of well-known classification methods. First, we demonstrate the feasibility of using a training dataset semi-automatically obtained from other accurate algorithms. In addition, we investigate the effect of ancillary information, e.g., surface type or climate, on accuracy. Then we compare the different classification methods using the same training dataset under different configurations. We also perform a consensus analysis aimed at estimating the degree of mutual agreement among classification methods in detecting Clear or Cloudy sky conditions.

## 1. Introduction

Despite the large existing literature, cloud detection from images taken by sensors onboard satellites is still an area of very active research. This is essentially due to three main reasons: (i) Cloud detection is an important preliminary step of remotely sensed image processing because clouds affect sensor measurements of radiance emitted by surface up to make data unreliable for a wide range of remote-sensing applications that use optical satellite images; (ii) Cloud detection by itself is a difficult problem (even for experts attempting to visually detect clouds from signatures and/or images) in some conditions as transparent or semi-transparent clouds and in general when the contrast between the cloud and the underlying surface is poor; and (iii) Despite consolidated guidelines for cloud detection algorithms (e.g., use of infrared bands, preliminary removal of noninformative bands), development of new sensors with different hardware capabilities in terms of spatial, spectral and temporal resolution claims for specific algorithms or adaption of existing ones (e.g., re-estimate of new thresholds). In particular, a dramatic progress in the technology was availability of hyper-spectral sensors able to take images up to 8K bands [1,2]. While these instruments promise to gather unprecedented information from surface and atmosphere, however, they challenge low-dimensional conventional algorithms for cloud detection not so much for scalability and computational resources required but as for physical and theoretical implications of hyper-spectrality. In particular, really informative bands must be selected in advance to face the curse of dimensionality inherent in statistical estimation (dimensionality reduction). Actually, a manual selection of spectral bands and estimate of thresholds for bands themselves or some couples of theirs is not conceivable anymore, therefore innovative methods for automatic feature extraction from the hyper-spectral images are sought.

In the case of low-dimensionality, as in PROBA-V considered in the present paper, the dimension reduction problem is generally not a concern and all spectral bands are considered (Experiments not reported in this paper confirm that best accuracy is achieved when all PROBA-V spectral bands are considered). However, due to the limited amount of information with a so reduced number of bands, it is important to extract relevant features for cloud detection as effectively as possible.

In all cases, also considering the recent explosive emerging methods, a problem of validation of methods arises that could help not only in comparing their accuracy but also to understand strengths and weaknesses of the general cloud detection problem.

Classification exercises are sometimes organized where different algorithms are challenged to estimate a cloud mask from radiance detected by a specific sensor. Radiance is endowed with labels on the Clear or Cloudy condition accurately assigned by experts that are blind to the algorithms, so to be used as a validation of the algorithms themselves. While the main purpose of such exercises is to develop accurate operational algorithms for specific sensors onboard satellites, an important side effect is comparison of state-of-art methods on a same, very accurate dataset. In this respect we mention the Landsat comparison exercise [3] and the ESA Round Robin exercise for PROBA-V sensor [4]. Such comparisons are an exceptional way not only to compare algorithms, but especially to discover their weakness in particular climatic/surface conditions and, finally, to progress knowledge of cloud mask detection.

One of the authors participated in the ESA Cloud Detection Round Robin exercise (https://earth.esa.int/web/sppa/activities/instrument-characterization-studies/pv-cdrr). Such exercise was intended for the PROBA-V sensor onboard the PROBA ESA platforms and suited for land use and classification, including vegetation, crop monitoring, food security and scarcity prediction, disaster and biosphere monitoring. PROBA-V has a small number of spectral bands (Blue, Red, NIR and SWIR) and in particular it lacks a Thermal Infrared band that could have been useful to detect cirrus clouds. This makes cloud detection from its images challenging. ESA and the Belgian Science Policy Office organized a dedicated Round Robin exercise to inter-compare the performances of different cloud detection algorithms for PROBA-V. The Round Robin exercise provided the participants with a large dataset of PROBA-V images (331 for almost 8 Billion scenes) covering all seasons, most surface types, different world zones and most cloud types (In this paper, we shall refer to pixel as the single element of the image matrix provided by the sensor with its field of view, including the corresponding geographical coordinates, and to scene as the set of corresponding information for that pixel, namely spectral radiance, sky condition, surface, climatic zone). The key data of the exercise is a set of 1350 scenes, blind to participants that have been manually labelled by experts, claimed to sample the most important types of clouds (*gold* standard). A more detailed description of the exercise and main conclusions are reported in [4].

The framework proposed by the authors for the Round Robin exercise includes a statistical classification method (Cumulative Discriminant Analysis, CDA [5]), a training set semi-automatically obtained from cloud masks estimated for concurrent sensors, and grouping data in almost homogeneous surface types. In particular, our framework was the only one within the exercise that did not use a manual dataset obtained by expert annotation to train the classification. Instead it was relying on a semi-automatic training obtained as the result of consolidated and acknowledged as reliable cloud masks with comparable spatial resolution as the target cloud mask (MODIS and SEVIRI). The only intervention required is spatial and temporal co-registration of the training cloud mask with the target cloud mask. In view of the fact that this training cloud mask is not obtained by expert judgement but by another algorithm, it will be defined as a *silver* standard. On the one hand quality of the silver standard cannot be compared with the accuracy of a gold standard. However, in this respect we also recall that even very accurate cloud masks annotated by experts are affected by judgement error that in best cases is estimated around 4–7% [3]. On the other hand, the much larger size of the training dataset and its wide coverage can represent a much larger number of cloud and surface conditions. This result cannot be obtained by manual training, naturally limited by human resources. This strategy appears in our opinion as a natural path when methods requiring large training datasets are involved. It is mainly the case of deep learning algorithms. In this respect we mention [6] who use the results of an algorithm (CFMask [3]) to train their deep RS-Net model for Landsat 8 images, and [7] based on AVIRIS cloud mask. Massive use of such a silver standard dataset for cloud detection was pioneered in [8] in our knowledge.

Another qualifying part of our framework was grouping of scenes into homogeneous zones selected basing on the surface type. It is frequent that algorithms for cloud detection are trained separately for different types of the underlying surface (e.g., land or water); other approaches are possible, for example introducing climatic information as in [5].

Aim of the present paper is first to show a full and detailed analysis of our framework and of the results within the Round Robin exercise, assessing its performance under several cloud and surface conditions.

In addition the availability of a very accurate gold standard allows one to quantitatively analyze weaknesses and strengths of cloud detection under a framework in which also the same training dataset is shared among classification methods. In particular, we address the following questions: (a) to compare prototypes of selected cloud detection algorithms well known in the literature; (b) to assess the feasibility of a silver standard to train cloud detection; (c) to assess the role of surface and/or climatic information on the accuracy of cloud detection. Finally, a consensus analysis is performed aimed at estimating the degree of mutual agreement among classification methods in detecting Clear or Cloudy sky conditions.

## 2. Data and Methods

### 2.1. PROBA-V Data

PROBA-V (PRoject for On-Board Autonomy-Vegetation) is a global vegetation monitoring mission [9], launched in 2013 to assure the succession of the Vegetation instruments onboard the French SPOT-4 and SPOT-5 Earth observation missions. The satellite follows a Sun-synchronous orbit at a height of 820 km, achieving a daily global coverage, except the equatorial region (within 35${}^{\circ }$ of the Equator) where coverage is guaranteed every two days. The optical instrument onboard provides from 1/3 km to 1 km-resolution data products. It captures a Blue band (centered at 463 nm), a Red band (centered at 655 nm), a Near-Infrared band (centered at 845nm), and a Short-wave Infrared band (centered at 1600 nm). The data of the traditional Vegetation products, as provided by PROBA-V, are freely accessible for all users. The new, higher resolution products of PROBA-V elder than 1 month share the same full, free and open data policy. Details on the technical characteristics of PROBA-V of interest for the present work are reported in Table 1.

We consider as input data 331 images released by ESA and provided by the organizers of the Round Robin exercise. These images are PROBA-V Level 2A products with Top-of atmosphere reflectance (the four PROBA-V bands radiometrically and geometrically corrected and resampled at 333 m). They conform a complete globe acquisition from four different dates covering the four seasons in 2014. PROBA-V scenes are endowed with a sea/land mask and an algorithm for snow/ice detection. The total number of valid scenes available in the 331 files is 7,731,538,861, the remaining ones being off sensor view, sun glint or missing reflectance.

### 2.2. Validation Dataset (Gold Standard)

All participants of the Round Robin exercise for PROBA-V cloud detection algorithm were provided with a small dataset consisting of 1350 scenes, manually collected, classified and labelled by an expert user. Since obtained by an expert, the dataset can be fully defined as a gold standard. The scenes are a subset of the full PROBA-V dataset collected from 4 different images and labelled with the following categories: Clouds (totally Cloudy, opaque clouds; semi-transparent clouds; other turbid atmosphere, e.g., dust, smoke); Clear sky (over water; snow/ice; other cases); spatially mixed clouds (over land; water; ice).

**Figure 1:**
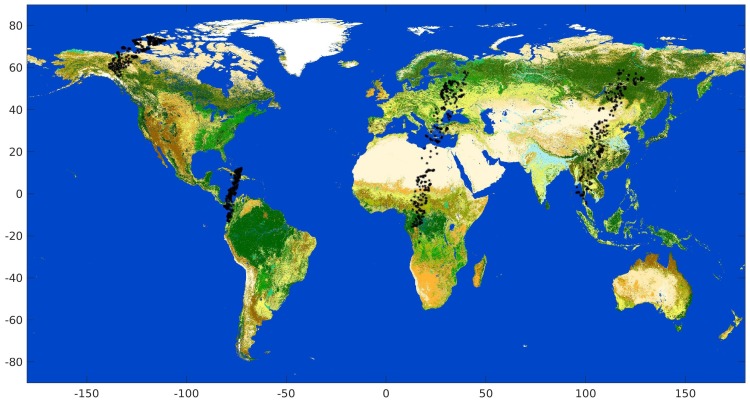
Global surface map as provided by GlobCover (22 classes). The superimposed black dots represent the pixels belonging to the gold standard dataset in the PROBA-V Round Robin exercise.

This small dataset comprises 30% totally Cloudy, 32% semi-transparent and 38% Clear cases. The relationship between land and water scenes is about 70:30 (land:water). The detailed distribution of categories is reported in Table 2. Figure 1 shows the world distribution of the dataset.

We mention that a second dataset of 53,000 scenes was prepared during the ESA project. It served as a basis for comparing algorithms participating in the Round Robin exercise [4]. However, this dataset has never been revealed to participants, even after the end of the exercise [10].

### 2.3. Training and Validation Dataset

To produce the training and validation sets in the PROBA-V exercise needed by the classification methods we rely on the cloud masks provided by consolidated algorithms endowed with SEVIRI and MODIS data. We assign labels to all the PROBA-V scenes for which both algorithms can provide labels, as reported in the following in detail.

#### 2.3.1. SEVIRI Cloud Mask

Processing data from SEVIRI (Spinning Enhanced Visible and Infrared Imager) sensor onboard MSG satellites provides a cloud mask dataset at 15 min temporal resolution [11] with a spatial resolution of 3Km sub-satellite that degrades far from the equator and from the Greenwich meridian. The data are provided with regional coverage within a radius of about 60 degrees around the point at zero latitude and longitude. No data are provided for the hemisphere including Americas, Oceania and most Asia. The grid of the SEVIRI cloud mask is 3712 × 3712 pixels; the number of valid cloud mask pixels is about 12M.

**Figure 2:**
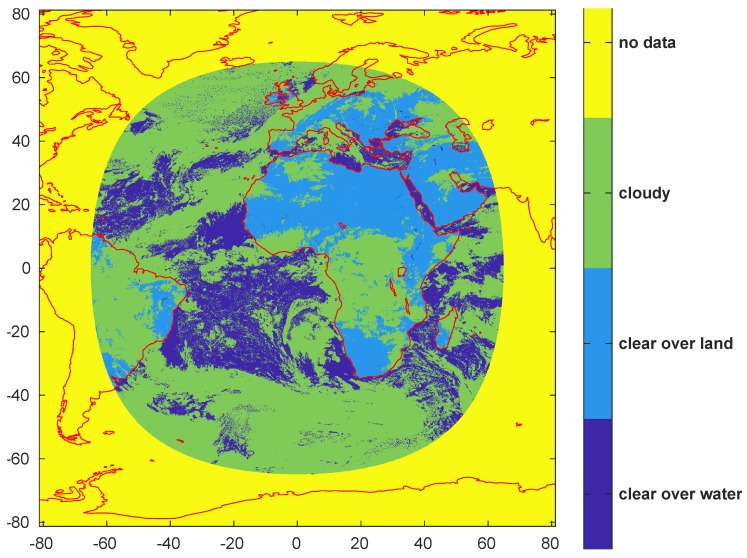
Example of SEVIRI cloud mask.

SEVIRI cloud mask provides four different labels: Clear over sea, Clear over land, Cloudy, uncertain. We considered for the training set only Clear and Cloudy SEVIRI scenes, so to represent the distribution of Clear and Cloudy conditions more accurately without influence of the other conditions. Moreover, since SEVIRI and PROBA-V scenes need to be co-located, we resampled the SEVIRI grid of pixels to a uniform grid in latitude and longitude. This choice preserves the original space resolution close to the center of the SEVIRI images. Of course, far from the center the finer resolution of the new grid is fake and the SEVIRI cloud mask is simply repeated within the coarser grid. Technically the procedure is equivalent to a Nearest Neighbor interpolation. Then we include in the training dataset only the PROBA-V scenes for which the closest SEVIRI pixel with respect to time and space provides a certain (i.e., Clear or Cloudy) label. Figure 2 shows an example of SEVIRI cloud mask.

#### 2.3.2. MODIS Cloud Mask

MODIS (MODerate-resolution Imaging Spectroradiometer) sensor flies onboard Earth Observation System Terra and Aqua satellites on polar orbits. There are two MODIS Cloud Mask L2 data product files: MOD35 [12], containing data collected from the Terra platform, and MYD35 [13], with data collected from the Aqua platform. The MODIS cloud mask provides fifteen classes (such as confident Clear over different surface types; possibly Clear; uncertain Clear; Cloudy) that provide basic surface and cloud type information.

**Figure 3:**
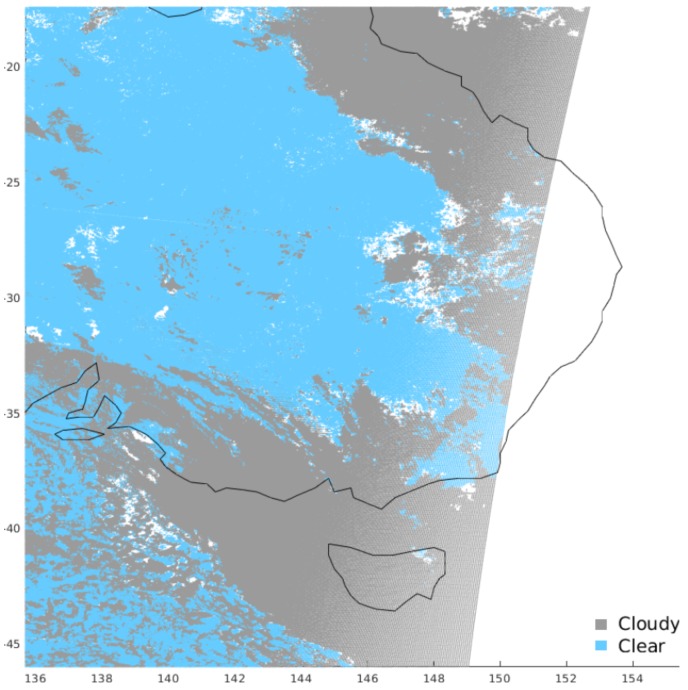
Example of MODIS cloud mask co-located to PROBA-V image PROBAV_L2A_20140321_000512_3_333M_V001 over South Australia.

We selected all the MODIS images for the four days of the PROBA-V exercise at 1Km spatial resolution. Their latitude and longitude grid is not uniform and it is given at a coarser resolution than the PROBA-V grid. For this reason, the grid of pixels is first interpolated to the full resolution of the sought PROBA-V cloud mask. Moreover, since the grid of the MODIS images depends on the granule and therefore is not fixed in time or space, we co-located the coarser MODIS grid into the PROBA-V uniform grid. Since MODIS orbits are polar, there is not always a good match in time with a PROBA-V pixel; we considered to be simultaneous only pixels whose acquisition differs at most by 30 min. We tested indeed shorter overlaps of time intervals (15 min and 7 min), and obtained comparable accuracy in the results. Clearly, the interpolation procedure can generate mixed/uncertain labels on some scenes in the finer grid. However, as already done for the SEVIRI cloud mask, we included in the training and validation set only PROBA-V scenes co-located to MODIS ones labelled as Cloudy and confident Clear. Figure 3 shows an example of MODIS cloud mask.

### 2.4. Combined SEVIRI and MODIS Cloud Mask

A single cloud mask is obtained from the SEVIRI and MODIS cloud masks of Section 2.3.1 and Section 2.3.2 by selecting all scenes for which there was an agreement of both. This choice enhances purity of the Clear and Cloudy classes for the purpose of classification. Since MSG points at 0${}^{\circ }$ longitude, it can take images only of Europe, Africa and part of Asia, therefore the merged database completely misses Americas, almost full Asia and Oceania for which several scenes of the gold standard are present (see Figure 1). The full joint SEVIRI and MODIS dataset is composed of 65M scenes, of which 40% labelled as Clear and 60% as Cloudy. This proportion is consistent with the gold standard in Table 2 (62% Cloudy scenes and 38% Clear ones). An extensive analysis through experiments analogous to the ones described in this paper (not reported for the sake of brevity) confirms that accuracy of the combined cloud mask outperforms the single ones. In [4] some comparisons are shown for the gold standard dataset.

### 2.5. Surface Type

Among the ancillary information that can improve cloud mask detection, a significant contribution can be given by the characterization of the underlying surface. To sort scenes into homogeneous regions with respect to their spectral behavior, one can use the sea/land mask endowed with PROBA-V scenes and an algorithm for snow/ice detection. Moreover, other and more detailed surface classifications can be considered, such as the GlobCover surface map. GlobCover [14] is a 2005 ESA initiative jointly with the Joint Research Center of the European Commission, the European Environment Agency, the Food and Agriculture Organization of the United Nations, the United Nations Environment Program, the Global Observation of Forest and Land Cover Dynamics and the International Geosphere-Biosphere Program. The aim of the project is to provide land cover maps from the 300 m MERIS sensor onboard the ENVISAT satellite mission. We consider the map that covers the period January–December 2009. The GlobCover map is provided as an image 55,800× 129,600 pixels in an equispaced grid with range [−65${}^{\circ }$,80${}^{\circ }$] for latitude and [−180${}^{\circ }$,180${}^{\circ }$] for longitude. The map sorts surface into 22 different classes with proper codes; a sample map is shown in Figure 1. To retain the most significant surface characteristics while avoiding unnecessary details, we group the GlobCover surface classes into the following five types: Water, Vegetation, Bare Land, Urban, Snow/Ice. They are shown in Table 3 together with their numerosity and percentage of Clear and Cloudy scenes both in the full training dataset and in the gold standard one.

### 2.6. Climate

We also consider grouping of the scenes into homogeneous climatic zones as in [5]. Specifically, we discriminate several regions based on latitude (Tropics, Mid and High Latitude); season (Winter, Summer); Hemisphere. We also rely on information from Elevation and Surface Temperature maps, as obtained from NASA (ASTER Global Digital Elevation Model, [15]) and the European Center for Medium-Range Weather Forecast (ERA-5 land hourly data from the Copernicus Climate Data Store, [16]) public databases, respectively. The specification of the climatic regions we consider is reported in Table 4. Due to the limited world coverage of the SEVIRI data, the number of climatic zones is less than [5] because some of them are empty.

## 3. Classification Methods

Cloud detection can be formally considered to be a binary supervised classification problem. As such, methods for its solution need a representative set of data with labels considered to be “certain” (training dataset). They evaluate patterns in different features and assign data into one of the two classes (Clear or Cloudy). The classification procedure also involves collection and evaluation of a validation dataset. Once trained, each classifier applies a decision rule to determine if validation data are more likely to have originated from one class or another. This rule partitions the *n*-dimensional feature space into 2 regions corresponding to the Clear and Cloudy conditions.

Operational cloud-masking algorithms on low/moderate-resolution sensors such as AVHRR and MODIS were mainly based on empirically tuned thresholds from several spectral channels. Also, for higher spectral resolution sensors (Landsat), thermal channel-based spectral thresholding along with prior knowledge of land surface properties has been the most common approach for automatic cloud detection. However, the payload of several recent sensors does not include thermal channels in which cloud-masking strategies have previously relied, so that different approaches, somewhat relying on complementary information have been pursued. Among the many methods and available implementations from the recent literature, we mention here a few significant ones. Taravat et al. [17] propose a Multilayer Perceptron for automatic classification of SEVIRI MSG images trained on the cloud mask by the European Organization for the Exploitation of Meteorological Satellites; Chen et al. [18] implement a neural network classifier driven by extensive radiative transfer simulations and validate it through collocated CALIOP and MODIS data. In [19], a Support Vector Machine classifier is trained on the Gabor energy characteristics of cloud superpixels from GF-1 images, while in [20] algorithms for Sentinel-2 MSI focused on Decision Trees and classical Bayesian classification are considered. Sedano et al. [21] propose a method based on the estimation of Clear/Cloudy radiance density distributions in a data fusion framework, followed by a region growing process and validate their results against both cloud masks generated by statistical methods and Landsat operational cloud mask. Finally, deep learning methods are increasingly considered, with several different approaches: in [22] a multi-modal, pixel-level Convolutional Neural Network-based classifier is introduced for detecting clouds in medium- and high-resolution remote-sensing images, which relies on a large number of per-pixel cloud masks digitized by experts; Francis et al. [23] use multi-scale features, based on a Fully Convolutional Network architecture, and report results on manually annotated images from two high-resolution sensors. An image-based approach is described in [24], relying on multi-modal, high-resolution satellite imagery (PlanetScope, Sentinel-2) at the scene level. Many of these methods rely on expert intervention for labelling training data. As mentioned in the Introduction, we focus instead on automatic means of assigning labels to the training and validation datasets (silver standard), allowing for adjustments of decision boundaries independent of subjective and costly human intervention. In addition, they can cover more general cases than manually possible ones and with a much larger extent, of course to the detriment of the decreased accuracy of labels.

Among the different approaches reported in the literature, we consider and compare for the present study seven different supervised classifiers. They fall into the categories usually labelled as Statistical and Machine Learning and are based on different principles, as Discriminant Analysis, Neural Networks, Nearest Neighbor. We mention that Neural Networks are the basis of Artificial Intelligence methods of present strong interest when the number of features is very high. In the following we briefly describe them.

1. Linear Discriminant Analysis (LDA). It applies the Bayes rule to each scene to select the Clear/Cloudy class so to maximize the posterior probability of the class for a scene given the actual reflectance in that scene. LDA assumes that reflectance follows Gaussian distributions for the Clear and Cloudy classes sharing the same covariance matrix;
2. Quadratic Discriminant Analysis (QDA), which generalizes LDA assuming that also covariance matrix depends on the class (Clear or Cloudy);
3. Principal Component Discriminant Analysis (PCDA) [25]: the hypothesis of Gaussian distribution of reflectance is released in favor of a generic distribution estimated by nonparametric regression; in addition the original reflectances are transformed into uncorrelated Principal Components before classification;
4. Independent Component Discriminant Analysis (ICDA) [25]: similar to PCDA, but with the original reflectances transformed into Independent Components before nonparametric estimation of the densities; this makes such components independent also for non-Gaussian distributions;
5. Cumulative Discriminant Analysis (CDA) [5]: the decision rule for classification depends on a single threshold for each feature (spectral band), based on the empirical distribution function, which discriminates scenes belonging to the Clear and Cloudy classes; the threshold is estimated so to minimize at the same time the false positive and false negative rates on the training or on a validation dataset.
6. Artificial Neural Networks (ANN) [26,27,28]. We use a two-layer feed-forward network, with sigmoid hidden and SoftMax output neurons for pattern recognition. The network is trained with scaled conjugate gradient backpropagation.
7. K-Nearest Neighbor (KNN) [28,29] that labels each considered scene based on a voting strategy among the labels assigned to the *K* closest neighboring scenes belonging to the training dataset. We used *K* = 50 throughout this study.

Methods LDA, QDA, PCDA, ICDA and CDA require estimate of the statistical distribution of radiance. We mention that other methods are available in the literature; results of some of them have not been reported because of poor accuracy on other sensors (Logistic Regression) or unfeasible computational time (Support Vector Machine).

All the above methods are pixel-wise, i.e., they treat pixels separately without taking account of spatial correlations among them or local features that are instead typical of images. Among the classification methods that use spatial features of images we mention [30] (Markov chains), [31] (Discriminant Analysis), [32] (relying on PCANet and SVM). We also mention the special case of Artificial Intelligence Deep Learning algorithms (e.g., [6,23]).

Finally, we mention that the method proposed for the Round Robin exercise was CDA [4].

## 4. Results

This section fully analyzes accuracy of the classification methods introduced in Section 3. Different configurations of classification are considered depending on the use of ancillary information.

### 4.1. Basic Classification

We consider as input data the reflectances of the scenes at the four spectral bands of PROBA-V. We extract a random training set of 3M scenes from the training dataset described in Section 2.3, whose cloud mask is assigned by the agreement of two consolidated algorithms (joint SEVIRI and MODIS silver standard). After the training phase, we classify the validation set composed of all the PROBA-V scenes for which a joint SEVIRI and MODIS cloud mask is available (65M scenes).

We remark that formally the training and validation datasets should be kept distinct, whereas the former is a subset of the latter. However, due to the large number of available scenes the features of the system are accurately learnt from the various methods in all conditions and are the same both in distinct and overlapping datasets. From the practical point of view all error indicators obtained keeping training and validation datasets distinct or overlapping have the same values up to 3 decimal digits. In addition, for the same reason a size of 3M scenes as a training set is sufficient for classification methods to accurately learn all the features of the data, so that no larger datasets are needed. In practice accuracy of the methods does not change when increasing the size of the training set even up to cover all the 65M available scenes. Using a smaller dataset for the training phase has the advantage of reducing computational time, which would otherwise be practically unfeasible for some methods such as ANN and KNN. Finally, we also mention that accuracy does not depend on the random choice of the 3M scenes subset within 3 decimal digits.

To estimate accuracy of the methods, we consider the following indicators: if $N_{\mathrm{Clear}}$ and $N_{\mathrm{Cloudy}}$ represent the number of Clear and Cloudy scenes in the validation dataset according to the MODIS and SEVIRI silver standard, respectively, and ${\hat{N}}_{\mathrm{Clear}}$ and ${\hat{N}}_{\mathrm{Cloudy}}$ the corresponding values estimated by any classification method, then we consider the global success accuracy *A*, $A_{\mathrm{Clear}}$ and $A_{\mathrm{Cloudy}}$ as

$$A=\frac{{\hat{N}}_{\mathrm{Clear}}+{\hat{N}}_{\mathrm{Cloudy}}}{N_{\mathrm{Clear}}+N_{\mathrm{Cloudy}}},\;A_{\mathrm{Clear}}=\frac{{\hat{N}}_{\mathrm{Clear}}}{N_{\mathrm{Clear}}},A_{\mathrm{Cloudy}}=\frac{{\hat{N}}_{\mathrm{Cloudy}}}{N_{\mathrm{Cloudy}}}.$$

Assuming Positive as the Cloudy condition, $A_{\mathrm{Clear}}$ is also known as Specificity (True Negative rate) and $A_{\mathrm{Cloudy}}$ as Sensitivity (True Positive rate).

Accuracy indicators *A*, $A_{\mathrm{Clear}}$ and $A_{\mathrm{Cloudy}}$ are shown in Table 5 for the entire dataset and the classification methods of Section 3; they are also sorted by the surface types introduced in Section 2.5. We remark that in this experiment the training and validation phases are both performed without using any surface information; surface sorting is performed after classification only to assess the accuracy level on different surface types.

As a complementary exercise, we compute accuracy indicators when the validation dataset is the gold standard of Section 2.2. The performance of all the algorithms is reported in Table 6. We advise that accuracy for some surfaces, namely Urban and Snow/Ice, is biased from the very low number of representative scenes and from lack of Clear or Cloudy scenes (compare with Table 3).

Analysis of results on the entire dataset (Table 5) shows first of all that ANN and KNN generally outperform methods based on the direct estimate of distributions, being the only ones to reach accuracies beyond 90% globally and in most cases. We also observe that CDA, which was used for the Round Robin exercise, is by far outperformed by ANN and KNN. This is mainly due to its very nature of forcing the same accuracy in both Clear and Cloudy conditions so to reach equal I- and II-Type errors globally (row Global in the table). This is paid when accuracy is assessed on a finer scale of surface type after classification has been made. As an example Bare Land has the most unbalanced proportion of Clear and Cloudy scenes, with latter ones being 8% of the total (see Table 3), therefore Clear scenes that globally have a lower frequency (40%) are strongly penalized. We also observe that accuracy is strongly dependent on the surface type for all methods; in particular it is higher on Land than on Water. This result is consistent among all methods participating the Round Robin exercise [4] and was there justified with a more accurate training. In this experiment this is equivalent to the fact that Land scenes are 2/3 of Total ones in the global training set, so that classification naturally tends to better represent them. On the other side main applications of PROBA-V sensor are for Land and vegetation, particularly.

Results are roughly consistent when we limit validation to the gold standard dataset for which the *true* Clear/Cloudy condition is known (Table 6). Accuracy is lower than the training set case by 7–8% for all methods. A noteworthy exception is CDA, whose global accuracy remains the same (actually, marginally better), even though no longer equibalanced between Clear and Cloudy conditions, the latter being increased to 87.1% at detriment of the former (74.4%). We again observe that ANN and KNN algorithms outperform methods based on the direct estimate of distributions, with accuracy rarely going beyond 90%, and that particularly for KNN is globally 2% higher than ANN, mostly due to a better detection of Cloudy condition (81.6% vs. 75.2%).

### 4.2. Use of Ancillary Information

Experiment of Section 4.1 considers the entire joint SEVIRI and MODIS dataset for the training (apart from the random subset selection). However, it is well known that capability of detecting clouds heavily depends on the underlying surface because of the different contrast between clouds and surface. Therefore, a possible useful strategy is to disaggregate the scenes into groups homogeneous as possible within, at a finer level than the land/water often used in operational cloud detection methods. For this purpose, we consider two possible disaggregations based on the type of surface and on the climate.

#### 4.2.1. Surface as Ancillary Information

We train the algorithms separately on different surface types. From the entire dataset we randomly extract a training set for each of the five surface types considered in Section 2.5. The size of these training sets is fixed as the minimum between 3M scenes and the subset size. Validation is performed on all the scenes of the same surface type in the full dataset. Table 7 reports the accuracy indicators. Table 8 refers to accuracy estimated using the gold standard as a validation dataset.

Comparison of Table 7 with Table 5 does not provide unique indications: most methods show a certain degree of decrease of accuracy around 1% globally (only LDA reaching −17%). Notable exceptions are ANN (+1%) and, especially, CDA (+5.8%). This is to be expected for CDA since the constraint of equal I- and II-Type errors now applies separately to each surface type and much better adapts to the frequency of Clear/Cloudy conditions that globally depends on the underlying type of surface. Improvement of CDA occurs for all surface types; in particular it is remarkable for that sky condition (Clear or Cloudy) with the worse accuracy when a unique classification was made without using ancillary information; however this improvement occurs in a less extent at detriment of the other sky condition (Cloudy or Clear, respectively).

If we compare Table 8 with Table 6 again we observe a drop in accuracy around 6–9% (now including CDA), somewhat smaller for KNN (4.4%).

Summarizing there is not a clear evidence from these quantitative indicators that separate classification problems tailored to different surface types improve accuracy. We will investigate further this matter later in Section 4.3.1 through visual inspection of the cloud masks provided by the different algorithms on some specific images.

#### 4.2.2. Climate as Ancillary Information

We train the classification methods separately on different climatic zones, as described in Section 2.6 and introduced in [5]. From the entire dataset a sample is extracted for each climatic zone as a training, with a size as the minimum between 3M and the subset size. As in Section 4.2.1 validation is performed on the entire dataset or on the gold standard one. Accuracy is shown in Table 9 and Table 10, respectively.

Also, this experiment does not provide a conclusive answer on the improvement of accuracy considering separate classifications for different climatic zones. Global increase of accuracy, when occurring (ANN among the best performing methods) is not spread over Clear and Cloudy conditions, rather a slight decrease in Clear conditions is observed. In the same way as separate classifications by surface type we observe a mix of better (ICDA and KNN) and worse (CDA and ANN) accuracies also spread over Clear and Cloudy conditions. The same conclusions can be drawn also on the gold standard dataset, with the remarkable better performance of CDA that is indeed the best performing method.

#### 4.2.3. Ancillary Information with ANN

Neural Networks are claimed to successfully mix information of different nature (e.g., nonnumeric). Our framework includes reflectance as a numeric information and surface type or climatic zone as a categorical variable. In Section 2.5 and Section 2.6 the categorical variable was dealt independently, in the sense that separate cloud masks were obtained with separate different training sets for each surface type or climatic zone. By its nature, ANN instead easily allows one to introduce the type of surface and/or climatic zone directly within the training dataset as a further fifth variable besides reflectance of the four spectral bands. The present Section aims at estimating if and how accuracy improves or degrades when compared with ancillary information used as separate classifications.

Results are shown in Table 11, where for the sake of brevity only Global results are shown, not disaggregated by type of surface or climate.

Comparison of Table 11 with Table 5, Table 7 and Table 9 shows that keeping separate classification for the different types of ancillary information improves accuracy of ANN.

### 4.3. Opaque and Semi-Transparent Clouds

This Section is intended to assess behavior of the considered classification methods with opaque and semi-transparent clouds and border identification. For this purpose, we consider two images already used in [4] and a quantitative analysis based on the gold standard dataset.

#### 4.3.1. Visual Analysis of Opaque and Semi-Transparent Clouds

**Figure 4:**
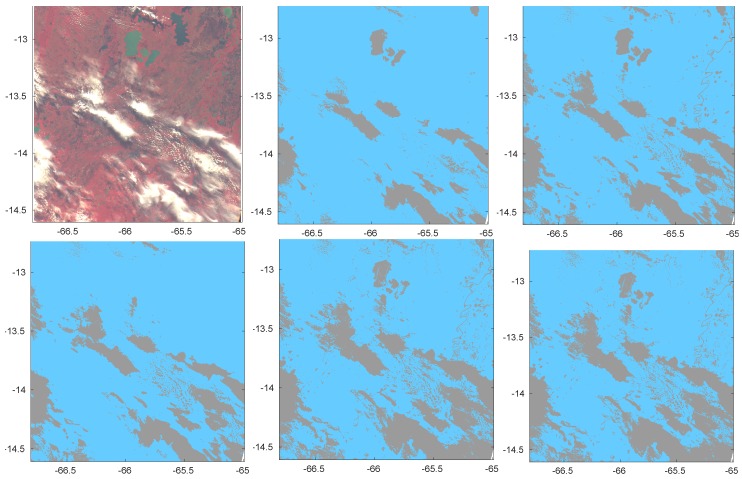
RGB image acquired on 21 June 2014 at 14:42 UTC over Bolivia (top-left panel). Other panels show cloud masks obtained by LDA, PCDA, CDA, ANN and KNN (orderly from left to right and from top to bottom). A single classification was made on the full joint training dataset SEVIRI and MODIS.

The first image was acquired on 21 June 2014 at 14:42 UTC over Bolivia overlying a land surface. It includes both opaque and semi-transparent clouds, as can be seen from the composite RGB image in the first panel of Figure 4. The other panels of the Figure show the cloud mask predicted by LDA, PCDA, CDA, ANN and KNN. All cases refer to the configuration of a unique classification independent of surface type or climate. An indirect comparison with other methods of the PROBA-V Round Robin exercise as reported in [4] can be made because the covered region is the same.

**Figure 5:**
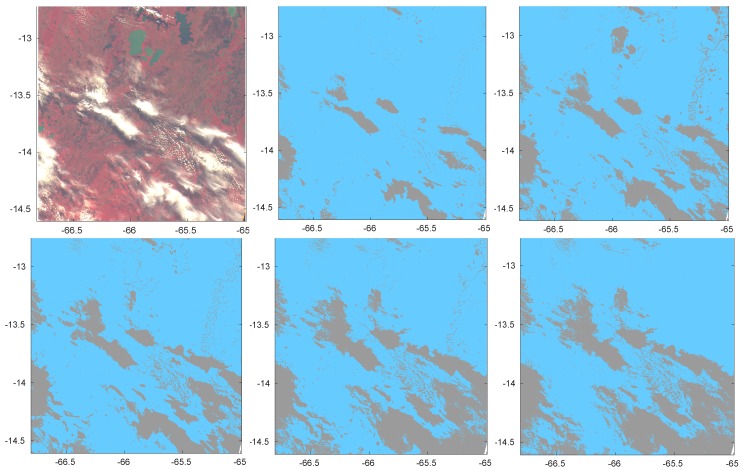
RGB image acquired on 21 June 2014 at 14:42 UTC over Bolivia (top-left panel). Other panels show cloud masks obtained by LDA, PCDA, CDA, ANN and KNN (orderly from left to right and from top to bottom). Classification was made separately for each type of surface.

Figure 5 shows the same results obtained when classification is made separately for each type of surface.

Figures clearly show that KNN and ANN are more cloud conservative than other methods. Despite the nonconclusive quantitative analysis on the role of separate classifications for different surface types or climatic zones, Figure 4 shows that a unique classification independent of surface or climate is prone to mistakes in interpreting Clear condition over water. Actually, some of the lakes in the Bolivia region (green color in the RGB image of the Figure) are misrecognized as Cloudy by 3 classification methods out of 5, including one of the best performing (ANN). On the contrary when classification is separately made by surface type only PCDA still misrecognizes some of the lakes as Cloudy, all other methods correctly detecting Clear sky conditions.

We also remark that KNN, though more conservative with respect to transparent clouds, is less prone to spurious Cloudy isolated scenes (see in particular the top-right part of Figure 5, bottom-right panel), while still preserving sharpness of the clouds.

**Figure 6:**
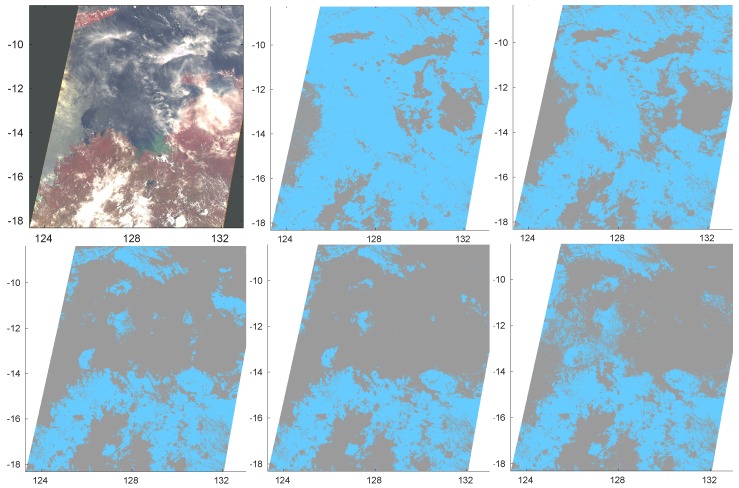
RGB image acquired on 21 December 2014 at 02:29 UTC over North Australia (top-left panel). Other panels show cloud masks obtained by LDA, PCDA, CDA, ANN and KNN (orderly from left to right and from top to bottom). Classification was made separately for each type of surface.

The second image, also considered in [4], was acquired on 21 December 2014 at 02:29 UTC over Northern Australia and includes both land and water surface. Figure 6 shows the RGB image and the cloud mask retrieved by LDA, PCDA, CDA, ANN and KNN (orderly from left to right and from top to bottom) when classification is made separately for each surface type.

The Figure confirms that ANN and KNN are more cloud conservative and semi-transparent clouds are detected as Cloudy.

#### 4.3.2. Quantitative Analysis of Semi-Transparent Clouds

To quantitatively analyze behavior of the classification methods with respect to opaque and semi-transparent clouds, Table 12 reports the percentage of semi-transparent scenes identified as Clear and Cloudy for all the considered methods. Table 12 quantitatively confirms the more conservative character of ANN and KNN throughout all the different experiments settings.

### 4.4. Consensus Analysis

This Section makes a consensus analysis aimed at assessing the mutual agreement of classification methods in estimating cloud mask. It can provide a further accuracy indicator, independent of any gold standard eventually available, and advice on the reliability of the cloud masks obtained by classification methods. Given a set of methods, we define consensus on Clear and Cloudy sky condition as the percentage of scenes for which all methods predict Clear and Cloudy conditions, respectively. The global consensus is defined as the direct sum of both Clear and Cloudy ones, and represents the percentage of scenes for which all methods agree in predicting the same sky condition, irrespective of the type, Clear or Cloudy. The percentage is computed on the entire set of 65M scenes of the dataset. We consider a subset of the classification methods considered in Section 3 according to the following guidelines: a) the methods have to be as independent as possible (however some degree of dependence cannot be avoided because all methods rely on the same training data set); b) the number of considered methods has to be low; in fact, the higher the number of methods, the higher probability that any of them predicts the status of the sky differently from the other ones, and therefore the lower the consensus. As a consequence we select only one representative method from the Discriminant Analysis group, namely PCDA that gives better accuracy in the analysis of Section 4.1, Section 4.2.1 and Section 4.2.2, keeping all the other methods (CDA, ANN, KNN). Should the number of methods be much larger, then a more elaborated definition of consensus should be devised.

First, we compute the consensus according to the results of Section 4.1 where a unique training dataset is considered independently of surface or climatic zone. The four methods agree on identifying 33% of the scenes as Clear and 49% as Cloudy, resulting in a full concordance on 82% of the scenes.

Then we consider the case when different separate classifications are performed for each type of surface (Section 2.5), whose results are reported in Table 13. When additional information on surface type is given, global concordance of the methods is slightly higher (83%), with a slightly better concordance on Clear scenes at detriment of Cloudy ones. Values disaggregated by surface type and for each sky condition are reported in the same Table: the highest consensus is obtained for Vegetation (87%) and the lowest one on Water (78%).

When separate classifications are made for each climatic zone, global consensus drops to 78%, mainly due to a lower consensus for Clear sky conditions (30.5%). Values for different climatic zones range from 73% (Tropical zone) to 89% (Mid Latitude Winter NH). Table 14 reports full results disaggregated by climatic zone and by sky condition.

Summarizing we can state that the highest consensus is reached when different classifications are made by surface type.

Finally, we investigate which of the selected classification methods deviates most from the consensus expressed from the other methods. For this purpose, we consider scenes for which only three of the 4 methods agree on the Clear or Cloudy condition and calculate the frequency of the methods that deviate from the other three ones.

In the basic classification (without additional information) the method that more often disagrees with the other three ones in predicting a Cloudy sky condition is PCDA. Specifically, when three methods out of four agree on Cloudy scenes, PCDA disagrees with them 41% of the times. When three methods agree in predicting a Clear sky condition, then CDA mostly disagrees (61% of scenes). The situation is similar when separate classifications are performed for each surface type (see row Global in Table 15): PCDA mostly disagrees with the other three methods (50.3% of scenes); this result is due to a very poor agreement in Cloudy sky conditions (disagreement in 87.9% of scenes), whereas in Clear sky conditions it is CDA that reaches the highest disagreement with 52.7% of scenes. This also occurs on the different surface types, with PCDA showing the highest disagreement for all types of surfaces but Bare Land, due to the highest disagreement in Cloudy conditions, whereas in Clear sky conditions CDA shows the highest disagreement on Vegetation and Bare Land, ANN on Water and Urban surface and KNN on Snow/Ice.

When classification is trained separately on different climate zones, as reported in Table 16, the situation is quite similar for PCDA and CDA, with ANN showing a greater disagreement in more cases.

Summarizing, we can say that PCDA and CDA show the highest disagreement with respect to the other methods and on the contrary KNN the lowest disagreement.

## 5. Conclusions

The paper shows a detailed analysis of the method presented by one the authors in a Round Robin exercise organized by ESA for detecting clouds from images taken by the PROBA-V sensor. Availability of a common high-quality dataset of scenes labelled as Clear or Cloudy by experts (gold standard) is a unique benchmark for comparing different methods for detecting clouds and investigating on questions still open.

We considered some prototypes of methods and compared them under different frameworks but using a common training dataset for all of them. We demonstrated that CDA, chosen for participating in the Round Robin, was adequate yielding good accuracy. However, ANN and, particularly, KNN can both improve accuracy and better detect scenes with semi-transparent clouds. In addition, a silver standard training dataset, semi-automatically obtained by algorithms developed for other sensors, was proved to be effective in detecting clouds, yielding high accuracy. We stress that the silver standard dataset considered in this paper covers only a portion of the globe and that the majority of gold standard pixels are outside the region covered by the silver standard. Indeed a silver standard dataset is the only feasible solution to get very large training datasets needed to train Artificial Intelligence methods (see, e.g., [7]).

Then, even though it was not possible to give a conclusive quantitative answer whether separate classifications based on ancillary information as surface types and/or climatic zones improve accuracy, a qualitative analysis shows that introducing such information reduces probability to misinterpret Clear/Cloudy condition on Water.

Finally, we performed a consensus analysis aimed at estimating the degree of mutual agreement among classification methods in detecting Clear or Cloudy sky. The result was that a selection of 4 classification methods agree on the status of Clear or Cloudy sky for about 83% of scenes. PCDA and CDA show the highest disagreement with the consensus of the other 3 methods and KNN the lowest disagreement. This result is consistent with the findings on accuracy.

Results shown in the paper strictly refer to sensors with a very low number of spectral bands. Other sensors, especially hyper-spectral, or at higher/lower spatial resolutions need a specific similar analysis that will probably give very different results.

Results of the paper suggest possible future investigations. a) The use of different methods for different surfaces or climatic zones: this study demonstrated that some methods could be more suited for particular surfaces or climatic zones when we consider global accuracy or specific accuracy for Clear or Cloudy conditions. b) Balancing of cloud/Clear conditions: this largely depends on the training dataset and on the fraction of Clear/Cloudy scenes included. Then the classification methods will generally and naturally favor the most populated class when Clear and Cloudy features overlap, so to improve global accuracy. However commonly remotely sensed images can refer to conditions that are prevalently Clear or prevalently Cloudy that could benefit from a training dataset or a classification method that weights scenes according to the different proportion of Clear/Cloudy conditions in the region. CDA is an attempt into this direction, even though in a global way, and Discriminant Analysis is naturally prone to include such weights, simulating a balancing of Clear and Sky conditions different from the training dataset. c) To use images instead of independent pixels in the classification, so to exploit spatial correlation (that clouds indeed possess) and/or equivalently spatial features. In this respect Artificial Intelligence methods, already available in the literature but not considered in this paper, become interesting also for a small number of spectral bands. 

## Figures and Tables

**Table 1:** Technical characteristics of PROBA-V sensor.

Characteristic	Value
Orbit	Sun-synchronous polar orbit, 98.73${}^{\circ }$ inclination, 820 km altitude
Coverage	35${}^{\circ }$–75${}^{\circ }$N and 35${}^{\circ }$–56${}^{\circ }$S daily 35${}^{\circ }$N–35${}^{\circ }$S every 2 day
Field of view	102${}^{\circ }$, 2250 km swath
Resolution	100 m at Nadir, 350 m full field of view
visual and infrared detector	
Blue band	447–493 νm
Red band	610–190 νm
Near-Infrared band	773–893 νm
Product resolution	1 km, 1/3 km
short-wave infrared detector	
Short-Wave Infrared band	1570–1650 νm
Product resolution	1 km, 2/3 km

**Table 2:** Distribution of the labelled scenes in the gold standard dataset provided to all the participants in PROBA-V Round Robin exercise.

Category	# Scenes	Percentage
Totally Cloudy	400	30%
Semi-transparent clouds	438	32%
**Total Cloudy scenes**	**838**	**62%**
Clear sky water	192	14%
Clear sky land	205	15%
Clear sky snow/ice	67	5%
Turbid atmosphere (no cloud) cases	23	2%
Other Clear cases	25	2%
**Total Clear scenes**	**512**	**38%**
Floating ice	67	5%
Glint	59	4%
Cloud shadow	46	3%
**Total**	**1350**	**100%**

**Table 3:** Surface types considered in the paper, their numerosity and percentage of Clear and Cloudy scenes in both the full joint MODIS and SEVIRI and the gold standard dataset.

	Full Dataset			*Gold* Standard Dataset		
Surface Type	# Scenes	Clear	Cloudy	# Scenes	Clear	Cloudy
Water	20,746,553 (31.8%)	19%	81%	326 (24.1%)	61%	39%
Vegetation	33,978,411 (52.1%)	37%	63%	921 (68.2%)	28%	72%
Bare Land	9,927,876 (15.2%)	92%	8%	83 (6.1%)	51%	49%
Urban	235,546 (0.4%)	24%	76%	9 (0.07%)	100%	0%
Snow/Ice	41,570 (0.06%)	39%	61%	10 (0.07%)	0%	100%
Total	65,238,704 (100%)	40%	60%	1350 (100%)	38%	62%

**Table 4:** Climatic zones considered in the paper and their numerosity in both the joint MODIS and SEVIRI dataset and the gold standard dataset.

	Full Dataset			*Gold* Standard Dataset		
Climatic Zones	# Scenes	Clear	Cloudy	# Scenes	Clear	Cloudy
Tropical	3,480,8034 (53.3%)	55%	45%	728 (53.9%)	34%	66%
Mid-Latitude Summer NH	18,610,636 (28.5%)	29%	71%	285 (21.1%)	42%	58%
Mid-Latitude Summer SH	270,858 (0.4%)	1%	99%			
Mid-Latitude Winter NH	8,173,055 (12.5%)	12%	88%			
Mid-Latitude Winter SH	573,416 (0.9%)	8%	92%			
High-Latitude Summer NH	2,240,391 (3.4%)	7%	93%	226 (16.7%)	39%	61%
Ice on sea NH	4950 (0.008%)	3%	97%	17 (1.3%)	24%	76%
Ice on sea SH	1804 (0.003%)	0%	100%			
Ice over land NH	343,163 (0.5%)	6%	94%	90 (6.7%)	60%	40%
Ice over land SH	1398 (0.002%)	0%	100%			
Ice over elevated land NH	210,958 (0.3%)	22%	78%	4 (0.3%)	0%	100%
Total	65,238,704 (100%)	40%	60%	1350 (100%)	38%	62%

**Table 5:** Accuracy indicators *A*, $A_{\mathrm{Clear}}$ and $A_{\mathrm{Cloudy}}$ of classification methods of Section 3 when validation is made on the full dataset and no ancillary information on surface or climate is used. Percentage values are reported for the entire dataset (row Global) and sorted for different surface types.

		LDA	QDA	PCDA	ICDA	CDA	ANN	KNN
	$A_{\mathrm{Clear}}$	70.7	96.4	91.5	94.2	95.0	81.5	87.1
Water	$A_{\mathrm{Cloudy}}$	81.5	68.0	77.3	74.2	75.9	94.2	96.1
	*A*	79.5	73.4	80.0	78.0	79.5	91.8	94.3
	$A_{\mathrm{Clear}}$	97.8	96.1	93.9	95.2	91.7	89.7	93.5
Vegetation	$A_{\mathrm{Cloudy}}$	69.8	79.2	85.2	83.4	87.2	91.3	91.7
	*A*	80.1	85.4	88.4	87.7	88.8	90.7	92.4
	$A_{\mathrm{Clear}}$	99.8	99.5	98.8	99.1	63.5	97.6	99.2
Bare Land	$A_{\mathrm{Cloudy}}$	54.6	68.5	72.8	70.7	88.8	76.3	74.3
	*A*	96.3	97.2	96.8	96.9	65.5	96.0	97.3
	$A_{\mathrm{Clear}}$	97.0	93.9	86.5	92.0	78.2	72.6	79.4
Urban	$A_{\mathrm{Cloudy}}$	76.7	83.7	88.8	86.8	90.5	94.1	93.6
	*A*	81.5	86.1	88.2	88.0	87.6	89.0	90.2
	$A_{\mathrm{Clear}}$	96.0	80.2	80.0	86.3	94.1	93.4	88.8
Snow/Ice	$A_{\mathrm{Cloudy}}$	59.0	65.6	67.1	68.9	59.4	81.2	93.8
	*A*	73.6	71.3	72.2	75.7	73.1	86.0	91.9
	$A_{\mathrm{Clear}}$	94.4	97.4	95.3	96.4	82.1	91.2	94.5
Global	$A_{\mathrm{Cloudy}}$	74.3	74.0	81.3	79.0	82.1	92.3	93.2
	*A*	82.2	83.2	86.9	85.9	82.1	91.9	93.8

**Table 6:** Accuracy indicators *A*, $A_{\mathrm{Clear}}$ and $A_{\mathrm{Cloudy}}$ of classification methods when validation is made on the gold standard dataset of Section 2.2 and no ancillary information on surface or climate is used. Percentage values are reported for the entire gold standard dataset (row Global) and sorted for different surface types.

		LDA	QDA	PCDA	ICDA	CDA	ANN	KNN
	$A_{\mathrm{Clear}}$	62.5	81.0	71.0	67.5	85.0	68.5	80.5
Water	$A_{\mathrm{Cloudy}}$	84.9	79.4	83.3	84.1	80.2	93.7	84.9
	*A*	71.2	80.4	75.8	73.9	83.1	78.2	82.2
	$A_{\mathrm{Clear}}$	68.6	68.2	65.5	64.8	64.8	77.4	80.8
Vegetation	$A_{\mathrm{Cloudy}}$	79.8	82.6	85.9	85.8	88.6	90.5	92.1
	*A*	76.7	78.5	80.1	79.8	81.9	86.8	88.9
	$A_{\mathrm{Clear}}$	90.5	85.7	78.6	81.0	78.6	88.1	88.1
Bare Land	$A_{\mathrm{Cloudy}}$	34.1	48.8	48.8	41.5	80.5	56.1	56.1
	*A*	62.7	67.5	63.9	61.5	79.5	72.3	72.3
Urban	$A_{\mathrm{Clear}}$	100	100	100	100	100	100	100
Snow/Ice	$A_{\mathrm{Cloudy}}$	100	100	100	100	100	100	100
	$A_{\mathrm{Clear}}$	68.6	75.2	69.3	67.8	74.4	75.2	81.6
Global	$A_{\mathrm{Cloudy}}$	78.6	80.7	83.9	83.5	87.1	89.4	89.4
	*A*	74.8	78.6	78.4	77.6	82.3	84.0	86.44

**Table 7:** Accuracy indicators *A*, $A_{\mathrm{Clear}}$ and $A_{\mathrm{Cloudy}}$ of classification methods of Section 3 when validation is made on the full dataset and classifications are separate for each surface type. Percentage values are reported for the entire dataset (row Global) and for the different surface types.

		LDA	QDA	PCDA	ICDA	CDA	ANN	KNN
	$A_{\mathrm{Clear}}$	97.7	94.5	92.2	93.4	89.0	80.8	92.8
Water	$A_{\mathrm{Cloudy}}$	68.1	71.4	75.7	75.4	89.0	96.2	93.2
	*A*	73.7	75.8	78.8	78.8	89.0	93.2	93.1
	$A_{\mathrm{Clear}}$	99.7	97.3	95.5	95.7	88.8	90.7	95.0
Vegetation	$A_{\mathrm{Cloudy}}$	48.4	74.4	81.3	80.7	88.8	91.9	90.1
	*A*	58.1	82.8	86.5	86.2	88.8	91.5	92.1
	$A_{\mathrm{Clear}}$	98.9	97.8	97.3	97.2	82.7	99.5	94.5
Bare Land	$A_{\mathrm{Cloudy}}$	62.1	76.2	78.2	79.4	82.7	71.3	89.5
	*A*	69.1	96.2	95.8	95.8	82.7	97.4	94.1
	$A_{\mathrm{Clear}}$	99.6	96.9	96.3	96.3	87.7	80.3	94.7
Urban	$A_{\mathrm{Cloudy}}$	50.6	78.0	80.7	81.1	87.7	92.0	87.8
	*A*	59.9	82.5	84.4	84.7	87.7	89.2	89.5
	$A_{\mathrm{Clear}}$	99.9	92.4	92.6	91.0	86.5	86.9	91.8
Snow/Ice	$A_{\mathrm{Cloudy}}$	32.8	74.1	74.4	84.6	86.5	89.1	94.4
	*A*	45.6	81.3	81.5	87.2	86.5	88.3	93.4
	$A_{\mathrm{Clear}}$	99.1	97.0	95.6	95.9	86.7	92.3	94.5
Global	$A_{\mathrm{Cloudy}}$	57.1	73.2	78.8	78.4	88.8	93.3	91.4
	*A*	64.8	82.6	85.5	85.3	87.9	92.9	92.7

**Table 8:** Accuracy indicators *A*, $A_{\mathrm{Clear}}$ and $A_{\mathrm{Cloudy}}$ of classification methods of Section 3 when validation is made on the gold standard dataset of Section 2.2 and classifications are separate for each climatic zone. Percentage values are reported for the entire dataset (row Global) and for the different surface types.

		LDA	QDA	PCDA	ICDA	CDA	ANN	KNN
	$A_{\mathrm{Clear}}$	90.0	73.0	63.5	61.5	73.0	70.5	91.0
Water	$A_{\mathrm{Cloudy}}$	77.8	81.7	84.9	84.1	86.5	93.7	86.5
	*A*	85.3	76.4	71.8	70.3	78.2	79.4	89.3
	$A_{\mathrm{Clear}}$	74.7	68.6	65.5	64.0	63.6	77.4	83.1
Vegetation	$A_{\mathrm{Cloudy}}$	78.2	80.3	85.0	85.6	90.3	92.3	91.8
	*A*	77.2	77.0	79.5	79.5	82.7	88.1	89.4
	$A_{\mathrm{Clear}}$	90.5	73.8	64.3	71.4	81.0	85.7	64.3
Bare Land	$A_{\mathrm{Cloudy}}$	48.8	58.5	58.5	58.5	65.9	56.1	63.4
	*A*	69.9	66.3	61.5	65.1	73.5	71.1	63.9
Urban	$A_{\mathrm{Clear}}$	100	100	100	100	100	100	100
Snow/Ice	$A_{\mathrm{Cloudy}}$	100	100	100	100	100	100	100
	$A_{\mathrm{Clear}}$	82.4	71.3	65.2	64.3	69.3	75.8	85.0
Global	$A_{\mathrm{Cloudy}}$	76.8	79.6	83.8	84.1	88.5	90.7	89.6
	*A*	79.0	76.4	76.7	76.6	81.3	85.0	87.9

**Table 9:** Accuracy indicators *A*, $A_{\mathrm{Clear}}$ and $A_{\mathrm{Cloudy}}$ of classification methods of Section 3 when validation is made on the full dataset and classifications are separate for each climatic zone of Section 2.6. Percentage values are reported for the entire dataset (row Global) and for the different climatic zones.

		LDA	QDA	PCDA	ICDA	CDA	ANN	KNN
	$A_{\mathrm{Clear}}$	90.6	98.0	95.6	96.1	75.7	93.0	95.0
Tropical	$A_{\mathrm{Cloudy}}$	80.3	70.6	81.3	78.3	76.0	89.1	93.6
	*A*	85.9	85.5	89.1	88.0	75.8	91.3	94.3
	$A_{\mathrm{Clear}}$	97.4	96.6	94.2	96.4	87.1	88.1	93.7
Mid-Lat Summer NH	$A_{\mathrm{Cloudy}}$	66.4	72.5	77.5	75.9	87.1	93.5	91.9
	*A*	75.5	79.6	82.4	81.9	87.1	91.9	92.4
	$A_{\mathrm{Clear}}$	100	98.9	98.6	98.6	94.9	95.3	98.3
Mid-Lat Summer SH	$A_{\mathrm{Cloudy}}$	85.8	88.9	91.3	91.7	94.3	97.8	96.9
	*A*	85.9	89.0	91.3	91.8	94.3	97.8	96.9
	$A_{\mathrm{Clear}}$	84.7	95.9	94.7	95.4	91.2	83.5	94.9
Mid-Lat Winter NH	$A_{\mathrm{Cloudy}}$	92.0	86.7	88.3	90.5	91.3	97.7	95.4
	*A*	91.1	87.8	89.1	91.1	91.3	96.0	95.3
	$A_{\mathrm{Clear}}$	17.2	99.1	99.0	98.8	98.2	80.9	93.8
Mid-Lat Winter SH	$A_{\mathrm{Cloudy}}$	98.5	75.8	78.8	83.0	83.5	97.3	94.9
	*A*	92.2	77.6	80.3	84.2	84.6	96.0	94.8
	$A_{\mathrm{Clear}}$	71.2	83.6	82.1	88.1	75.0	39.6	90.8
Hi-Lat Summer NH	$A_{\mathrm{Cloudy}}$	88.9	77.4	80.0	81.4	82.8	96.1	89.5
	*A*	87.7	77.8	80.2	81.8	82.3	92.2	89.6
	$A_{\mathrm{Clear}}$	0	87.7	77.4	96.1	89.0	78.7	97.4
Ice on sea NH	$A_{\mathrm{Cloudy}}$	99.3	98.2	98.2	92.6	98.8	99.9	93.6
	*A*	96.2	97.9	97.6	92.7	98.5	99.3	93.7
	$A_{\mathrm{Clear}}$	0	0	0	0	0	0	0
Ice on sea SH	$A_{\mathrm{Cloudy}}$	99.9	99.7	99.1	97.7	99.9	99.9	97.1
	*A*	99.9	99.7	99.1	97.7	99.9	99.9	97.1
	$A_{\mathrm{Clear}}$	19.0	26.0	24.1	35.0	20.0	25.9	74.6
Ice over land NH	$A_{\mathrm{Cloudy}}$	96.8	96.6	97.0	95.7	99.0	99.2	95.1
	*A*	92.1	92.3	92.6	92.0	94.2	94.8	93.9
	$A_{\mathrm{Clear}}$	0	0	0	0	0	0	0
Ice over land SH	$A_{\mathrm{Cloudy}}$	100	100	100	99.0	100	100	99.6
	*A*	100	100	100	99.0	100	100	99.6
	$A_{\mathrm{Clear}}$	35.4	42.1	42.4	54.1	35.3	22.4	77.9
Ice-over-elev-land NH	$A_{\mathrm{Cloudy}}$	95.6	95.7	95.8	91.9	98.0	99.2	91.4
	*A*	82.2	83.8	83.9	83.5	84.1	82.1	88.4
	$A_{\mathrm{Clear}}$	0	0	0	0	0	0	0
Ice-over-elev-land SH	$A_{\mathrm{Cloudy}}$	100	100	100	100	100	100	100
	*A*	100	100	100	100	100	100	100
	$A_{\mathrm{Clear}}$	91.4	97.4	95.0	96.0	78.6	91.1	94.6
Global	$A_{\mathrm{Cloudy}}$	78.7	75.0	81.4	80.2	83.3	92.8	93.2
	*A*	83.7	83.9	86.8	86.4	81.5	92.1	93.7

**Table 10:** Accuracy indicators *A*, $A_{\mathrm{Clear}}$ and $A_{\mathrm{Cloudy}}$ of classification methods of Section 3 when validation is made on the gold standard dataset of Section 2.2 and classifications are separate for each climatic zone of Section 2.6. Percentage values are reported for the entire dataset (row Global) and for the different climatic zones.

		LDA	QDA	PCDA	ICDA	CDA	ANN	KNN
	$A_{\mathrm{Clear}}$	64.8	86.2	68.0	68.4	88.7	72.5	78.9
Tropical	$A_{\mathrm{Cloudy}}$	86.5	85.2	87.9	86.9	89.8	90.2	91.5
	*A*	79.1	85.6	81.2	80.6	89.4	84.2	87.2
	$A_{\mathrm{Clear}}$	85.7	70.6	67.2	68.1	66.4	62.2	74.8
Mid-Lat Summer NH	$A_{\mathrm{Cloudy}}$	59.0	61.4	68.7	66.9	83.1	84.3	83.7
	*A*	70.2	65.3	68.1	67.4	76.1	75.1	80.0
	$A_{\mathrm{Clear}}$	86.4	87.5	84.1	75.0	83.0	40.9	75.0
Hi-Lat Summer NH	$A_{\mathrm{Cloudy}}$	89.1	95.7	95.7	90.6	89.9	100	89.9
	*A*	88.1	92.5	91.2	84.5	87.2	77.0	84.1
	$A_{\mathrm{Clear}}$	0	0	0	0	0	0	0
Ice on sea NH	$A_{\mathrm{Cloudy}}$	100	100	100	100	100	100	100
	*A*	76.5	76.5	76.5	76.5	76.5	76.5	76.5
	$A_{\mathrm{Clear}}$	90.7	100	100	94.4	94.4	74.1	90.7
Ice over land NH	$A_{\mathrm{Cloudy}}$	86.1	38.9	22.2	52.8	88.9	97.2	88.9
	*A*	88.9	75.6	68.9	77.8	92.2	83.3	90.0
	$A_{\mathrm{Clear}}$	0	0	0	0	0	0	0
Ice-over-elev-land NH	$A_{\mathrm{Cloudy}}$	100	100	100	100	75.0	100	100
	*A*	100	100	100	100	75.0	100	100
	$A_{\mathrm{Clear}}$	75.6	83.6	73.4	71.7	82.4	64.3	77.9
Global	$A_{\mathrm{Cloudy}}$	81.7	80.5	82.8	82.3	88.5	91.2	89.7
	*A*	79.4	81.7	79.3	78.3	86.2	81.0	85.3

**Table 11:** Accuracy of ANN when ancillary information is introduced as a fifth variable. Global accuracy is shown for the entire dataset (*A*) and separately for Clear ($A_{\mathrm{Clear}}$) and Cloudy ($A_{\mathrm{Cloudy}}$) scenes when no ancillary information are used, and surface type, climate and both are used.

	$A_{\mathrm{Clear}}$	$A_{\mathrm{Cloudy}}$	*A*
Only reflectances	91.2	92.3	91.9
With surface information	92.1	92.0	92.1
With climatic information	89.0	91.0	90.2
With both surface and climatic information	92.0	92.0	92.0

**Table 12:** Percentage of semi-transparent clouds detected as Clear or Cloudy by classification methods in the three classification configurations (no ancillary information; with surface information; with climatic information). The total number of semi-transparent scenes in the gold standard dataset is 438.

	No Ancillary Information		Surface		Climate	
	Clear	Cloudy	Clear	Cloudy	Clear	Cloudy
LDA	39	61	77	23	34	66
QDA	37	63	39	61	35	65
PCDA	31	69	31	69	32	68
ICDA	32	68	30	70	32	68
CDA	25	75	22	78	22	78
ANN	20	80	18	82	17	83
KNN	20	80	20	80	20	80

**Table 13:** Global Consensus of PCDA, CDA, ANN, KNN (last row) and separately on different surface types when different separate classifications are performed for each surface type.

	Clear	Cloudy	Total
Water	17.26	60.78	78.04
Vegetation	35.35	51.92	87.27
Bare Land	72.86	5.85	78.71
Urban	23.94	61.57	85.51
Snow/Ice	34.30	40.51	74.81
Global	35.59	47.49	83.08

**Table 14:** Global Consensus of PCDA, CDA, ANN, KNN (last row) and separately on different climatic zones when different separate classifications are performed for each climatic zone. Only zones with sample size of the training set larger than 1M scenes are shown.

	Clear	Cloudy	Total
Tropical	39.54	33.91	73.45
Mid-Latitude Summer NH	27.12	54.84	81.96
Mid-Latitude Winter NH	11.26	77.32	88.58
High-Latitude Summer NH	5.79	71.75	77.54
Global	30.55	47.61	78.16

**Table 15:** Deviation of each classification method from the consensus expressed by other ones in the case classification is made separately for each surface type. Figures report the fraction of times a method disagrees with the other three ones that agree on the sky status. The higher the figure, the higher deviation from the consensus. Methods showing the highest disagreement for each surface type and sky conditions are indicated as boldface.

Surface	Clear Sky				Cloudy Sky				Total			
	PCDA	CDA	ANN	KNN	PCDA	CDA	ANN	KNN	PCDA	CDA	ANN	KNN
Water	9.62	3.02	**84.42**	2.93	**93.23**	2.27	0.19	4.31	**73.41**	2.45	20.16	3.98
Vegetation	1.82	**51.17**	19.73	27.27	**84.67**	1.04	0.96	13.33	**52.49**	20.51	8.25	18.75
Bare Land	6.30	**75.75**	0	17.95	0.81	5.93	**91.83**	1.43	6.06	**72.71**	4.00	17.23
Urban	1.90	12.01	**79.03**	7.06	**87.77**	3.27	1.66	7.30	**44.69**	7.66	40.48	7.18
Snow/Ice	18.72	26.45	19.89	**34.94**	**67.54**	21.21	4.19	7.06	**59.09**	22.12	6.91	11.88
Global	5.57	**52.72**	23.96	17.75	**87.92**	1.86	2.19	8.03	**50.31**	25.09	12.13	12.47

**Table 16:** Deviation of each classification method from the consensus expressed by other ones in the case classification is made separately for each climatic zone. Figures report the fraction of times a method disagrees with the other three ones that agree on the sky status. The higher the figure, the higher deviation from the consensus. Methods showing the highest disagreement for each climatic zone and sky conditions are indicated as boldface.

Climatic	Clear sky				Cloudy sky				Total			
zone	PCDA	CDA	ANN	KNN	PCDA	CDA	ANN	KNN	PCDA	CDA	ANN	KNN
Tropical	3.23	**73.09**	8.34	15.34	16.82	**73.10**	4.32	5.76	6.20	**73.09**	7.46	13.25
Mid-Latitude Summer NH	9.42	40.47	**41.04**	9.07	**94.46**	0.44	0.80	5.30	**40.44**	16.59	17.03	6.82
Mid-Latitude Winter NH	3.99	10.07	**77.89**	8.05	**77.34**	4.21	2.34	16.11	39.04	7.27	**41.78**	11.91
High-Latitude Summer NH	0.05	1.24	**91.03**	7.69	39.42	10.02	0.27	**50.29**	19.88	5.66	**45.31**	29.15
Global	3.96	**66.01**	16.92	14.10	**52.24**	35.73	2.57	9.45	19.56	**55.56**	12.29	12.60

## Abbreviations

The following abbreviations are used in this manuscript:

ANN	Artificial Neural Network
ASTER	Advanced Spaceborne Thermal Emission and Reflection Radiometer
AVHRR	Advanced Very-High-Resolution Radiometer
AVIRIS	Airborne Visible/InfraRed Imaging Spectrometer
CALIOP	Cloud-Aerosol LiDAR with Orthogonal Polarization
CDA	Cumulative Discriminant Analysis
ERA-5	ECMWF Reanalysis 5th Generation
ESA	European Space Agency
ICDA	Independent Component Discriminant Analysis
KNN	*k*-Nearest Neighbor
LDA	Linear Discriminant Analysis
MERIS	MEdium Resolution Imaging Spectrometer
MODIS	MODerate-resolution Imaging Spectroradiometer
MSG	Meteosat Second Generation
MSI	MultiSpectral Instrument
NASA	National Aeronautics and Space Administration
NH	Northern Hemisphere
NIR	Near-InfraRed
PCDA	Principal Component Discriminant Analysis
PROBA-V	PRoject for On-Board Autonomy – Vegetation
QDA	Quadratic Discriminant Analysis
SEVIRI	Spinning Enhanced Visible and Infrared Imager
SH	Southern Hemisphere
SPOT	Satellite Pour l’Observation de la Terre
SWIR	Short-Wave InfraRed
UTC	Coordinated Universal Time
